# Combined Analysis of Pharmaceutical Active Ingredients and Transcriptomes of *Glycyrrhiza uralensis* Under PEG6000-Induced Drought Stress Revealed Glycyrrhizic Acid and Flavonoids Accumulation *via* JA-Mediated Signaling

**DOI:** 10.3389/fpls.2022.920172

**Published:** 2022-06-13

**Authors:** Hua Yao, Fei Wang, Quan Bi, Hailiang Liu, Li Liu, Guanghui Xiao, Jianbo Zhu, Haitao Shen, Hongbin Li

**Affiliations:** ^1^Key Laboratory of Xinjiang Phytomedicine Resource and Utilization of Ministry of Education, Key Laboratory of Oasis Town and Mountain-basin System Ecology of Xinjiang Production and Construction Corps, College of Life Sciences, Shihezi University, Shihezi, China; ^2^Department of Pharmacology, Institute of Materia Medica of Xinjiang, Urumqi, China; ^3^Institute for Regenerative Medicine, Shanghai East Hospital, Tongji University School of Medicine, Shanghai, China; ^4^Cotton Institute, Xingjiang Academy of Agricultural and Reclamation Science/Northwest Inland Region Key Laboratory of Cotton Biology and Genetic Breeding, Shihezi, China; ^5^College of Life Sciences, Shaanxi Normal University, Xi’an, China

**Keywords:** *Glycyrrhiza uralensis* transcriptome, drought stress, UHPLC-MS/MS, glycyrrhizic acid, flavonoids, jasmonic acid

## Abstract

*Glycyrrhiza uralensis* contains many secondary metabolites with a wide range of pharmacological activities. Drought stress acts as a positive regulator to stimulate the production of medicinal active component in *G. uralensis*, however, the underlying mechanism remains unclear. The aim of this work is to investigate the accumulation and regulatory mechanism of pharmaceutical active ingredients in *G. uralensis* under drought stress. The materials of the aerial and underground parts of *G. uralensis* seedlings treated by 10% PEG6000 for 0, 2, 6, 12, and 24 h were used for RNA sequencing and determination of phytohormones and pharmaceutical active ingredients. PEG6000, ibuprofen (IBU), and jasmonic acid (JA) were utilized to treat *G. uralensis* seedlings for content detection and gene expression analysis. The results showed that, the contents of glycyrrhizic acid, glycyrrhetinic acid, and flavonoids (licochalcone A, glabridin, liquiritigenin, isoliquiritigenin, and liquiritin) were significantly accumulated in *G. uralensis* underground parts under drought stress. Kyoto Encyclopedia of Genes and Genomes analysis of the transcriptome data of drought-treated *G. uralensis* indicated that up-regulated differentially expressed genes (UDEGs) involved in glycyrrhizic acid synthesis in the underground parts and flavonoids synthesis in both aerial and underground parts were significantly enriched. Interestingly, the UDEGs participating in jasmonic acid (JA) signal transduction in both aerial and underground parts were discovered. In addition, JA content in both aerial and underground parts under drought stress showed the most significantly accumulated. And drought stress stimulated the contents of JA, glycyrrhizic acid, and flavonoids, coupled with the induced expressions of genes regulating the synthesis and transduction pathway. Moreover, In PEG6000- and JA-treated *G. uralensis*, significant accumulations of glycyrrhizic acid and flavonoids, and induced expressions of corresponding genes in these pathways, were observed, while, these increases were significantly blocked by JA signaling inhibitor IBU. JA content and expression levels of genes related to JA biosynthesis and signal transduction were also significantly increased by PEG treatment. Our study concludes that drought stress might promote the accumulation of pharmaceutical active ingredients *via* JA-mediated signaling pathway, and lay a foundation for improving the medicinal component of *G. uralensis* through genetic engineering technology.

## Introduction

*Glycyrrhiza uralensis* is an important medicinal plant for its roots as key ingredient in traditional Chinese medicine that contains many components with a wide range of pharmacological activities, such as anti-inflammatory (Bai et al., 2020), antidepressant (Dhingra and Sharma, 2006), antiviral (Fiore et al., 2008), hepatoprotective (Li et al., 2009), and anticancer (Aipire et al., 2017) properties. Glycyrrhizic acid and liquiritin have been known to play a role in the prevention and treatment of the new coronavirus pneumonia (Chai et al., 2020; Zhu et al., 2020a,b). Presently, cultivated licorice is the main source for commercial utilization, however, a higher planting cost is generated to make the pharmaceutical active ingredients reaching the standard, thus, attempts to decrease the cost by evaluating the contents or reducing the planting period have been performed comprehensively. Understanding the synthesis of the active ingredients of *G. uralensis* and underlying the regulatory mechanism will aid in improving quality and developing the use of the medicinal (underground parts of roots and rhizomes) and nonmedicinal (aerial parts of stems and leaves) materials for application in medicine, food, chemical industry, and other fields.

Glycyrrhizic acid and flavonoids are the major pharmacological active ingredients in *G. uralensis*, which are mainly found in underground parts (roots and rhizomes) and constitute up to 8% of the total dry weight of the plant (Afreen et al., 2005; Hayashi and Sudo, 2009). Glycyrrhizic acid is synthesized mainly *via* the cytosolic mevalonic acid (MVA) pathway, and many genes involved in this synthesis have been successfully cloned and characterized, including the 3-hydroxy-3-methylglutaryl-coenzyme A reductase gene (*HMGR*; Chappell et al., 1995; Li et al., 2017, 2020a,b; Mochida et al., 2017), squalene synthase gene (*SQS*; Li et al., 2017), squalene epoxidase gene (*SQLE*; Haralampidis et al., 2002), β-amyrin synthase gene (*β-AS*; Mochida et al., 2017), cytochrome P450 monooxygenase gene (*CYP88D6* and *CYP72A154*; Seki et al., 2008, 2011), and uridine diphosphate (UDP)-glycosyltransferases gene (*UGT*; Nomura et al., 2019). Liquiritin, licochalcone A, liquiritigenin, isoliquiritigenin, and glabridin are the main types of flavonoids in *G. uralensis*, among which liquiritin is the major type with a high preponderant concentration. The biosynthesis of flavonoids in plants begins with the phenylpropane metabolic pathway (Weisshaar and Jenkins, 1998; Dixon et al., 2002); chalcone synthase (CHS) and chalcone isomerase type II (II-CHI) are the key rate-limiting enzymes in the liquiritin synthesis pathway (Shimada et al., 2003; Yin et al., 2019). The synthetic pathway of glabridin is generally predicted to be through isoflavone synthesis (Simmler et al., 2013); however, the exact mechanism is still unclear.

Moderate drought stress significantly promoted the accumulation of pharmaceutical active ingredients in *G. uralensis* (Li et al., 2011), the important physiological roles of glycyrrhizic acid and flavonoids against biotic and abiotic stresses in *G. uralensis* were also been reported (Afreen et al., 2005; Hayashi and Sudo, 2009), showing the close link between drought stress and pharmaceutical active ingredients. Phytohormones are considered to play important role in mediating plant defense response against abiotic stresses (Bari and Jones, 2009; Nakashima and Yamaguchi-Shinozaki, 2013). Among them, jasmonic acid (JA) and abscisic acid (ABA) regulated the synthesis of flavonoids and triterpenoids by activating MYC2 transcription factors during drought and salt stress (Sreenivasulu et al., 2012).

Currently, key genes, metabolic pathways, and regulatory mechanism associated with the biosynthesis and regulation of pharmaceutical active ingredients in *G. uralensis* in response to drought stress are largely unknown. In this study, on the basis of the *G. uralensis* seedlings treated by PEG6000-induced drought stress for different times, combined analyses of UHPLC-MS/MS and RNA-sequencing (RNA-seq)-based transcriptome were performed to analyze the levels of endogenous hormones, pharmaceutical active ingredients, and the expressions of related genes in relevant biosynthesis pathways. We conclude that PEG6000-induced drought stress might promote accumulation of pharmaceutical active ingredients *via* JA-mediated signaling pathway. Our study also provides an effective reference for molecular mechanism elucidation of plants in response to drought stress, and lays a solid foundation to supply important key genes controlling synthesis of pharmaceutical active ingredients for improvement of quality of *G. uralensis* medicinal component through genetic engineering technology.

## Materials and Methods

### Plant Materials and PEG6000 Treatment

The seeds of *G. uralensis* were treated with 98% concentrated H_2_SO_4_ for 50 min to break the seed dormancy, washed with sterilized distilled water three times, and disinfected with 0.1% HgCl for 10 min. Treated seeds were cultured in a modified Hoagland solution in an automatic climate chamber under a steady condition (200 μmol mm^−2^ s^−1^ light intensity, 16 h light/8 h dark photoperiod, 50–55% relative humidity, and 28°C/25°C day/night culture temperature). The 60-day-old *G. uralensis* seedlings were transferred to Hoagland solution medium with 10% PEG6000 for stress treatment. The underground and aerial parts were collected, respectively, after 0, 2, 6, 12, and 24 h of continuous PEG6000 treatment. Throughout the sampling period, water was supplied regularly to keep the concentration of PEG6000. Ibuprofen (IBU) was used as the inhibitor of JA synthesis (Ankala et al., 2009). *Glycyrrhiza uralensis* were pretreated with 5 mM IBU for 1 h before PEG6000 treatment. For JA treatment, exogenous 50 μM JA was sprayed to the aerial parts of *G. uralensis* seedlings. The underground parts and aerial parts were collected after 2 and 6 h, under respective continuous treatments of PEG6000, mix of PEG6000 and IBU, and JA. Water supplementation was used as control (CK). All samples of collected aerial and underground parts were washed three times with sterile water, and were then quickly frozen in liquid nitrogen immediately after collection and stored at −80°C for further use. Three independent biological replicates were created for each treatment group, with 15 seedlings in each group.

### RNA Sequencing and Data Assembly

RNA from different materials of *G. uralensis* was extracted using Plant RNA Purification kit (TIANGEN, Beijing, China), according to the manufacturer’s instructions, which were then utilized as the template to synthesize cDNA for quantitative real-time PCR (qRT-PCR) assay. High-quality RNA samples were used to construct the sequencing library and validate the RNA-seq data. RNA Purification, reverse transcription, library construction, and sequencing were performed by Shanghai Majorbio Bio-pharm Biotechnology Co., Ltd. (Shanghai, China) using Illumina HiSeq 4000 platform according to the manufacturer’s instructions (Illumina, San Diego, CA). The obtained raw paired-end reads were trimmed and cleaned using SeqPrep^1^ and Sickleṅ^2^ with default parameters. The cleaned data were used for *de novo* assembly with Trinity.^3^ The online softwares of TopHat2^4^ and HISAT2^5^ were used to compare the clean data with the local downloaded *G. uralensis* genome.

### Screening and Functional Enrichment Analysis of Differentially Expressed Genes

Differentially expressed genes (DEGs) between control and treatment groups were identified using the Transcripts Per Million (TPM) method. The RNA-Seq by Expectation–Maximization (RSEM)^7^ was used to quantify gene and isoform abundances. The R statistical software package EdgeR^8^ was utilized for analyzing differential expression. Kyoto Encyclopedia of Genes and Genomes (KEGG) pathway analysis were analyzed by KOBAS.^9^

### Determination of Endogenous Phytohormones and Pharmaceutical Active Ingredients

All samples were washed three times with deionized water and were then quickly ground in liquid nitrogen. One gram fresh weight (FW) sample powder was added to a centrifuge tube with 5 ml 100% methanol and subsequently ultrasonicated (1,000 W) for 60 min at 25°C with subsequent centrifugation extract at 12,000 × *g* for 5 min at 4°C. The supernatants were collected and transferred into a new centrifuge tube for centrifugation of 12,000 × *g* for 5 min at 4°C. The extraction was performed twice, and all filtered extracts were collected into a 10-ml centrifuge tube to determine the pharmaceutical active ingredients. An appropriate amount of gibberellin A3 (GA_3_), licochalcone A, glabridin, ABA, brassinolide (BR), liquiritigenin, isoliquiritigenin, zeatin (ZT), JA, glycyrrhetinic acid, liquiritin, indole-3-acetic acid (IAA), and glycyrrhizic acid were accurately weighed and prepared into 1.0 mg/ml stock solutions that were then diluted with methanol to final concentration of 1, 5, 10, 25, 50, 75, and 100 ng/ml as standard solution to construct a standard curve using UHPLC-MS/MS (Supplementary Table S1). Separations were performed using a Waters ACQUITY UPLC BEH C18 column (50 mm × 5 mm, 1.7 μm, Waters, United States). The chromatographic conditions were as follows: temperature 30°C, flow rate 0.3 ml/min, and injection volume 1.0 μl. The final optimized mobile phase includes 0.1% formic acid water (A) and acetonitrile (B), with the gradient elution: 0–3.0 min, 20–98% B; 3.0–4.5 min, 98% B; 4.5–5.0 min, 98–20% B; 5.0–6.0 min, and 20% B. MS was performed in the multi-reaction monitoring (MRM) mode, with optimal conditions as follows: source temperature 200°C, capillary voltage 2,300 V, source offset 50 V, and desolvation temperature 450°C. The flow rate of desolvation gas and cone gas was 750 and 150 L/h, respectively. Waters MassLynx software (Version 4.1) was used for data acquisition and processing. MS parameters of each component are listed in Supplementary Table S2 and the total ion flow diagram of the detected substances is shown in Supplementary Figure S1.

### Validation of RNA-Seq Data Using qRT-PCR

To validate the DEGs identified from the RNA-seq data, eight DEGs that control carotenoid biosynthesis, phenylpropanoid biosynthesis, and hormone signaling were selected for qRT-PCR analysis with specific primers (Supplementary Table S3). The LightCycler 480 Real-Time PCR System (Roche Diagnostics International, Rotkreuz, Switzerland) was used for qRT-PCR assays, with *GuLectin* (Gene ID: Gu100s00008376) as the internal control for normalization. The relative expression values were determined by 2^−ΔΔ*C*t^ method, and a heatmap was generated by the MultiExperiment Viewer (MeV) version 4.8.0 (Dana-Farber Cancer Institute, Boston, MA).

### Statistical Analysis

SPSS statistics software 13.0 was used for result analysis, including descriptive statistics of data, correlation analysis of factors.

## Results

### Determination of Pharmaceutical Active Ingredients of *Glycyrrhiza uralensis* Under PEG6000 Treatment

**Figure 1:**
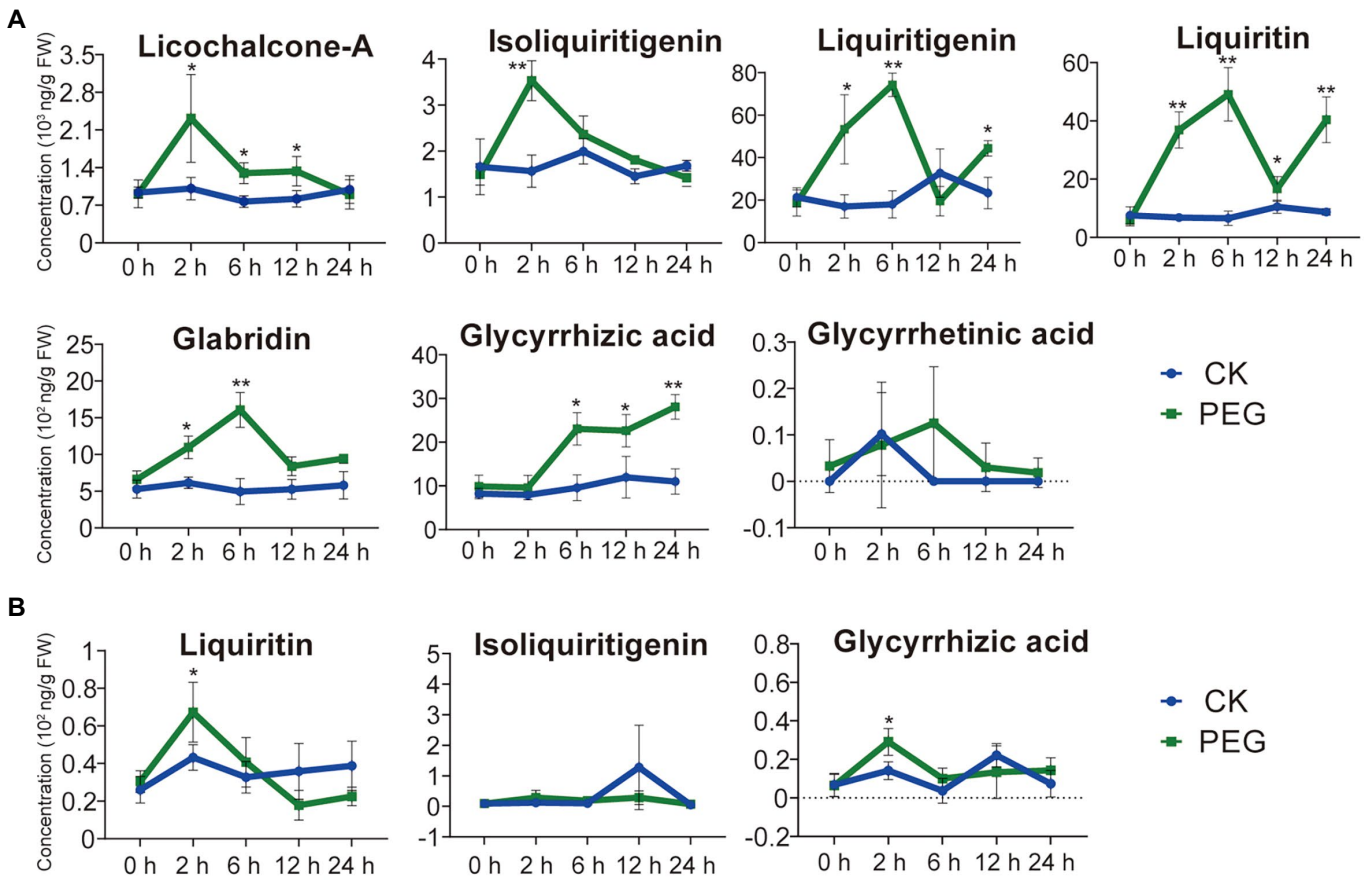
Content of seven pharmaceutical active ingredients of *Glycyrrhiza uralensis* under different durations of drought stress. **(A)** Levels of seven pharmaceutical active ingredients in the underground parts of *G. uralensis* under different durations of drought stress. **(B)** Levels of seven pharmaceutical active ingredients in the aerial part of *G. uralensis* under different durations of drought stress. The active ingredients, including glycyrrhizic acid, glycyrrhetinic acid, liquiritin, isoliquiritigenin, liquiritigenin, licochalcone-A, and glabridin, were measured in both aerial and underground parts of *G. uralensis*. Glycyrrhetinic acid, glabridin, licochalcone A, and liquiritigenin cannot be detected in aerials parts. Water addition was used as control (CK). Values are averages of the stress treatment group and control group (*n* = 15). ^*^p < 0.05, ^**^p < 0.01.

To understand the effect of different durations of drought stress on the pharmaceutical active ingredients in *G. uralensis*, we determined the contents of seven major ingredients including glycyrrhizic acid, glycyrrhetinic acid, liquiritin, isoliquiritigenin, liquiritigenin, licochalcone-A, and glabridin. Except for glycyrrhetinic acid, drought stress substantially stimulated the accumulations of the above pharmaceutical active ingredients in underground parts of *G. uralensis*, only two ingredients of liquiritin and glycyrrhizic acid in aerial parts showed slight increase (Figure 1). In *G. uralensis* underground parts, the contents of isoliquiritigenin and licochalcone-A indicated the highest levels in the 2-h stress group, the contents of liquiritin, liquiritigenin, and glabridin showed the highest levels in the 6-h stress group. The contents of liquiritin and glycyrrhizic acid displayed the most prominent levels. The level of liquiritin in the 6-h group was approximately 7.48-fold higher than that in the 0-h group. As a substrate of liquiritin biosynthesis (Yin et al., 2019), liquiritigenin content in the 6-h treatment group reached 74,240 ng/g FW, which was 4.11-fold higher than that in the 0-h treatment group. The level of glycyrrhizic acid increased significantly under drought stress, and showed a content of 28,093 ng/g FW that was 2.55-fold higher than that in the 0-h group (Figure 1A). In *G. uralensis* aerial parts, of the seven major ingredients, only glycyrrhizic acid, liquiritin, and isoliquiritigenin can be detected. The contents of glycyrrhizic acid and liquiritin indicated a slight increase in 2-h treatment group than that in in the 0-h treatment group (Figure 1B). These results demonstrate that drought stress significantly promotes the accumulation of pharmaceutical active ingredients in *G. uralensis* with a major effect in underground parts.

### Global Transcriptomic Changes of *Glycyrrhiza uralensis* During PEG6000-Induced Drought Stress

The genome-wide gene expression profile of *G. uralensis* subjected to PEG6000-induced drought stress treatment was analyzed by RNA sequencing (RNA-Seq), and the transcriptome changes at different treatment time points within 24-h of drought stress were analyzed. A total of 43.7–51.7 million reads in the underground parts and 42.9–47.7 million reads in the aerial parts were generated from the cDNA library for each treatment group (Supplementary Table S4). Hat2 software (version 2.1.0) at the website of http://ccb.jhu.edu/software/hisat2/index.shtml was used to compare the high-quality sequences of *G. uralensis* to, the reference genome, generating that 34.3–44.1 million reads in underground parts and 38.4–42 million reads in aerial parts were mapped to the genome of *G. uralensis*. Eight genes involved in terpene trunk biosynthesis, phenylpropane biosynthesis, and JA biosynthesis were verified by qRT-PCR, and the correlation analysis of qRT-PCR results and RNA-seq data showed high consistence (Supplementary Figure S2). Among the transcriptome data of each treatment group, more than 83.28% of clean reads could be uniquely mapped to the genome of *G. uralensis*. Among these expressed unigenes, 4,116 unigenes in the underground parts (Supplementary Figures S3A,B) and 5,087 unigenes in the aerial parts (Supplementary Figures S3C,D) were identified as differentially expressed genes (DEGs; *p*-adjust<0.05, |log_2_FC| > 2) between at least two different groups. STEM analysis indicated that 9 and 10 clusters in underground parts and aerial parts (Supplementary Figures S3E,F) were obtained, respectively. In which seven expression profiles were classified, and profiles I–IV including five clusters in underground parts and six clusters in aerial parts were identified as significantly up-regulated profiles.

### Kyoto Encyclopedia of Genes and Genomes Enrichment Analysis of Significantly Up-Regulated DEGs

**Figure 2:**
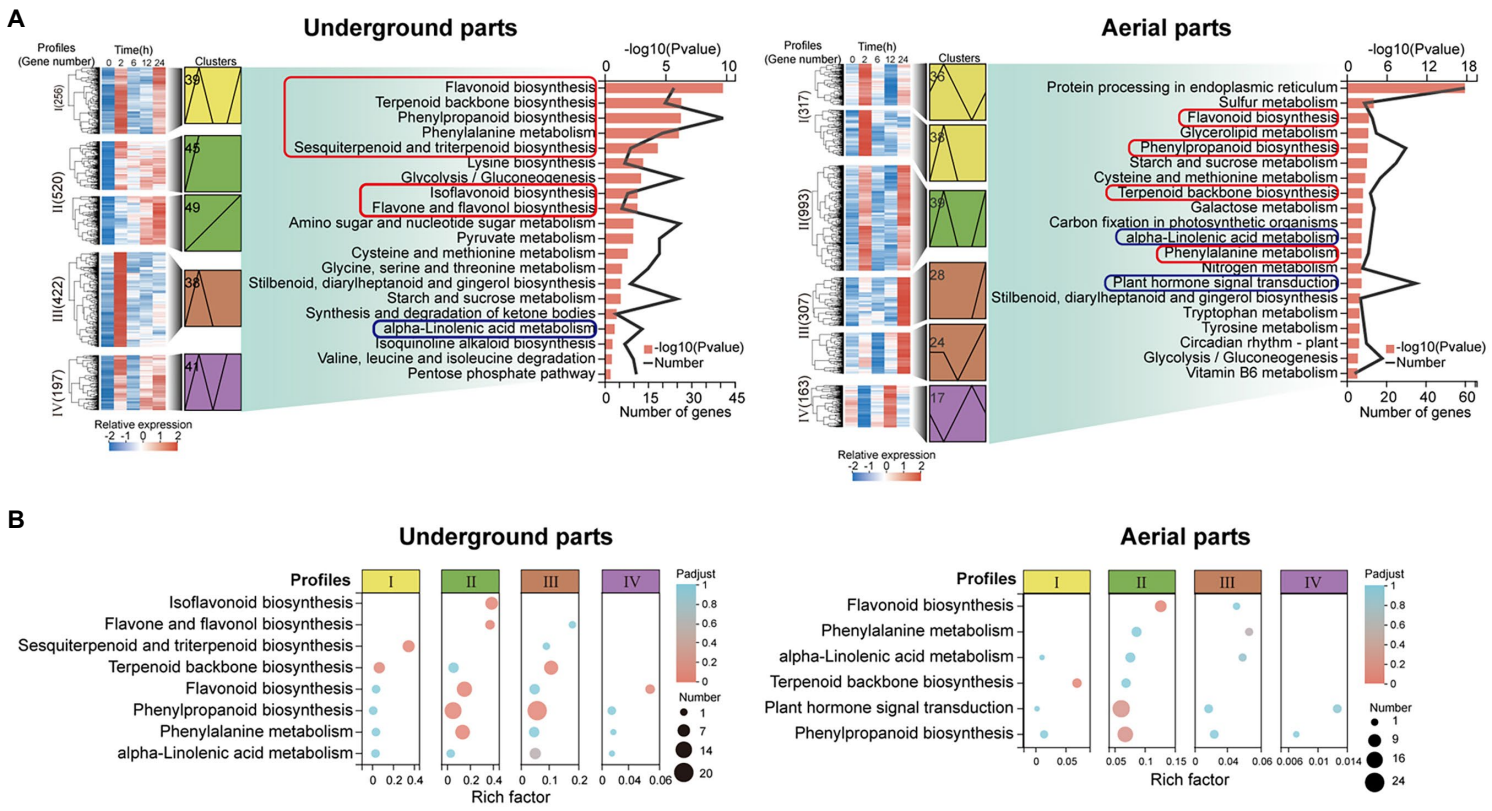
Transcript abundance analysis of up-regulated differentially expressed genes (UDEGs) of *Glycyrrhiza uralensis* under drought stress. **(A)** Kyoto Encyclopedia of Genes and Genomes (KEGG) enrichment analysis of the UDEGs of profiles I−IV in the aerial and underground parts of *G. uralensis*. The red and blue boxes represented the pathways of the synthesis of glycyrrhizic acid and flavonoids and of plant hormone synthesis and signal transduction, respectively. **(B)** Statistic analysis of UDEGs of profiles I−IV enriched in the pathways of the synthesis of glycyrrhizic acid and flavonoids and of plant hormone synthesis and signal transduction. Profiles I−IV were indicated by different color boxes and different size dots delegated the number of enriched UDEGs.

Kyoto Encyclopedia of Genes and Genomes enrichment analysis was performed on the up-regulated DEGs (UDEGs) of the underground parts and aerial parts to reveal their possible functions. Interestingly, of the most significant 20 enriched pathways, the pathways involved in the synthesis of pharmaceutical active ingredients of glycyrrhizic acid and flavonoids were discovered in both underground parts and aerial parts, including terpenoid backbone biosynthesis (path ID: map00900), sesquiterpenoid and triterpenoid biosynthesis (path ID: map00909), phenylalanine metabolism (path ID: map00360), phenylpropanoid biosynthesis (path ID: map00940), flavonoid biosynthesis (path ID: map00941), flavone and flavonol biosynthesis (path ID: map00944), and isoflavonoid biosynthesis (path ID: map00943; Figure 2, Supplementary Table S5). Remarkably, the pathways related to plant hormone signal and transduction were indicated, especially, the JA and ABA signal transduction pathways showed the most abundant enriched UDEGS (Figure 2, Supplementary Table S6). These results indicated the potential function of the UDEGs related to the synthesis of pharmaceutical active ingredients (glycyrrhizic acid and flavonoids) and of JA in the underground and aerial parts of *G. uralensis* in response to drought stress.

### Analysis of UDEGs Involved in Flavonoid Biosynthesis Pathways in *Glycyrrhiza uralensis* Under Drought Stress

**Figure 3:**
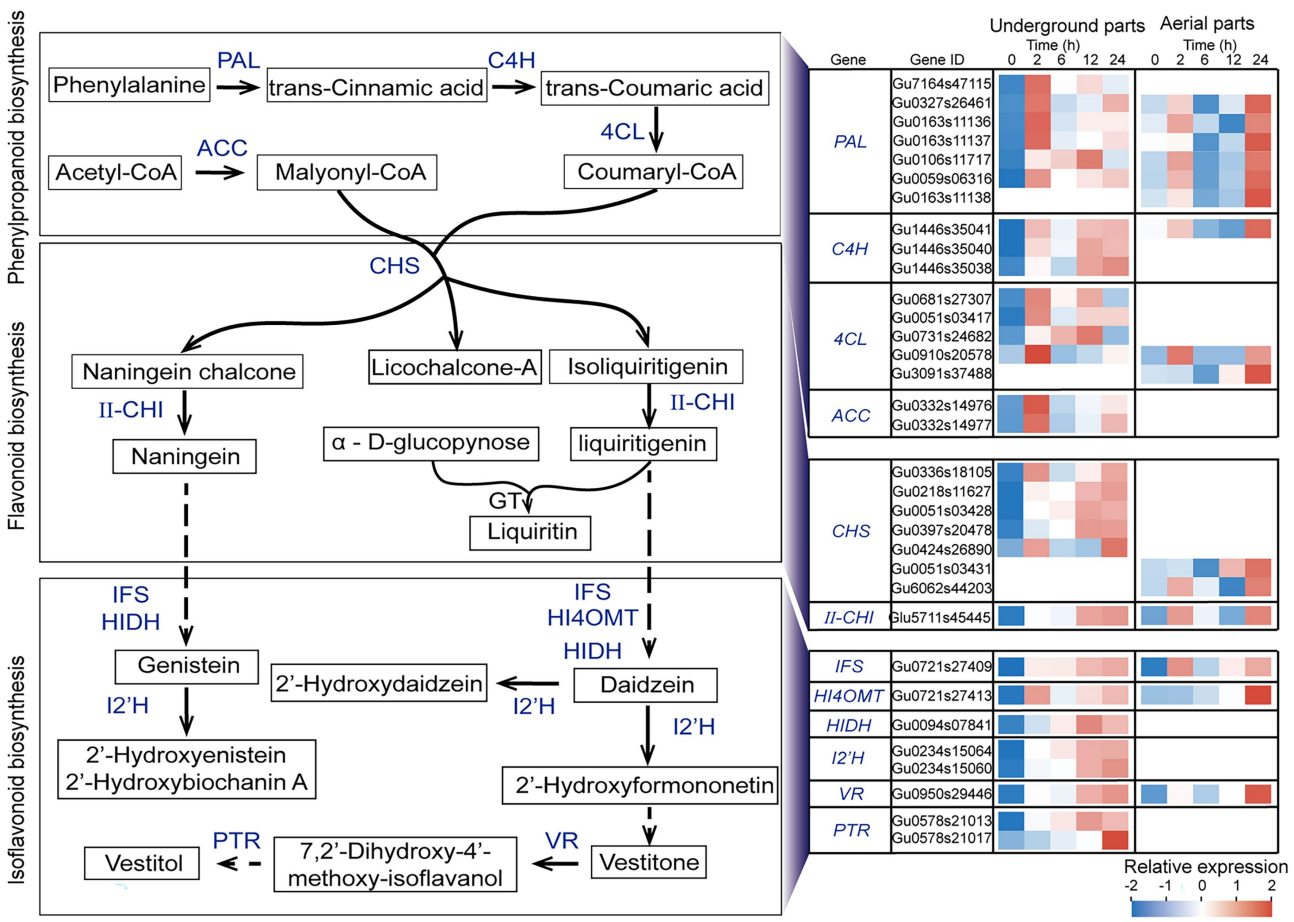
Expression analysis of up-regulated differentially expressed genes (UDEGs) involved in flavonoids biosynthesis pathway in *Glycyrrhiza uralensis* under drought stress. Blue words represent the identified UDEGs involved in flavonoids biosynthesis of *G. uralensis* under PEG6000-induced drought stress. Different colored blocks indicated different expression levels with red and blue to represent high and low levels, respectively. PAL, Phenylalanineammonialyase; C4H, Trans-cinnamate 4-monooxygenase; 4CL, 4-Coumarate-CoA ligase; ACC, Acetyl-CoA carboxylase; CHS, Chalcone synthase; II-CHI, II type Chalcone-flavonone isomerase; GT, Glycosyltransferase; IFS, Isoflavonoid biosynthesis; HI4OMT, Isoflavone 4’-O-methyltransferase; HIDH, 2-Hydroxyisoflavanone dehydratase; I2’H, Isoflavone 2′-hydroxylase; VR, Vestitone reductase; PTR, Pterocarpan reductase.

Considering the results that the flavonoids synthesis including phenylalanine metabolism, phenylpropanoid biosynthesis, flavonoid biosynthesis, and isoflavonoid biosynthesis were the most significant enriched pathways, in order to investigate the potential functions of these UDEGS, the expression features of all the relevant UDEGS were further analyzed in both aerial and underground parts of *G. uralensis* in response to drought stress (Supplementary Table S7). In the underground parts of *G. uralensis*, the UDEGs of *PAL*, *C4H*, *4CL*, *ACC*, and *CHS* that involved in phenylpropanoid and flavonoid biosynthesis showed prompt up-regulation expression after 2 h treatment of PEG6000 and maintained constant steady levels thereafter (Figure 3), with a over 24-fold increase expression of *CHS* (Gu0336s18105) in 2-h treatment group than that in 0-h control group. *II-CHI* (Glu5711s45445) displayed significant induced expression in 12-h and 24-h treatment groups with a 5-fold increase than that in 0-h control group.

CHS is considered to be the key enzyme in naningein and liquiritin synthesis. Five CHSs (Gu0336s18105, Gu0424s26890, Gu0218s11627, Gu0051s03428, and Gu0397s20478) showed specific expression in underground parts. The isoflavonoid biosynthesis (pathway ID: map00943) UDEGs containing *IFS*, *HI4OMT*, *HIDH*, *I’2H*, *VR*, and *PTR* demonstrated similar expression feature that showed significant enrichment expression in 12-h and 24-h treatment groups. In which *ACC*, *HIDH*, *I’2H,* and *PTR* indicated specific induced expression in underground parts but not in aerial parts. In the aerial parts of *G. uralensis*, six *PALs*, two *4CL* and *CHS*, one *C4H*, *IFS*, *HI4OMT*, and *VR*, were discovered as UDEGs involved in the biosynthesis of phenylpropanoid and flavonoids, and indicated consistent expression pattern with significant accumulation in 24-h treatment group (Figure 3). Seven CHSs were identified as UDEGS in *G. uralensis*, in which five in underground parts and two in aerial parts indicated tissue- and time-specific expression characteristics, respectively. The UDEGs locating in phenylpropanoid and flavonoids pathways showed more gene number and more fast expression response in underground parts than in aerial parts. These results demonstrated that flavonoids biosynthesis UDEGs had different expression feature in underground and aerial parts with main accumulation in underground parts, and might perform diverse potential functions in response to drought stress.

### Analysis of UDEGs Involved in Glycyrrhizic Acid Biosynthesis Pathway in *Glycyrrhiza uralensis* Under Drought Stress

**Figure 4:**
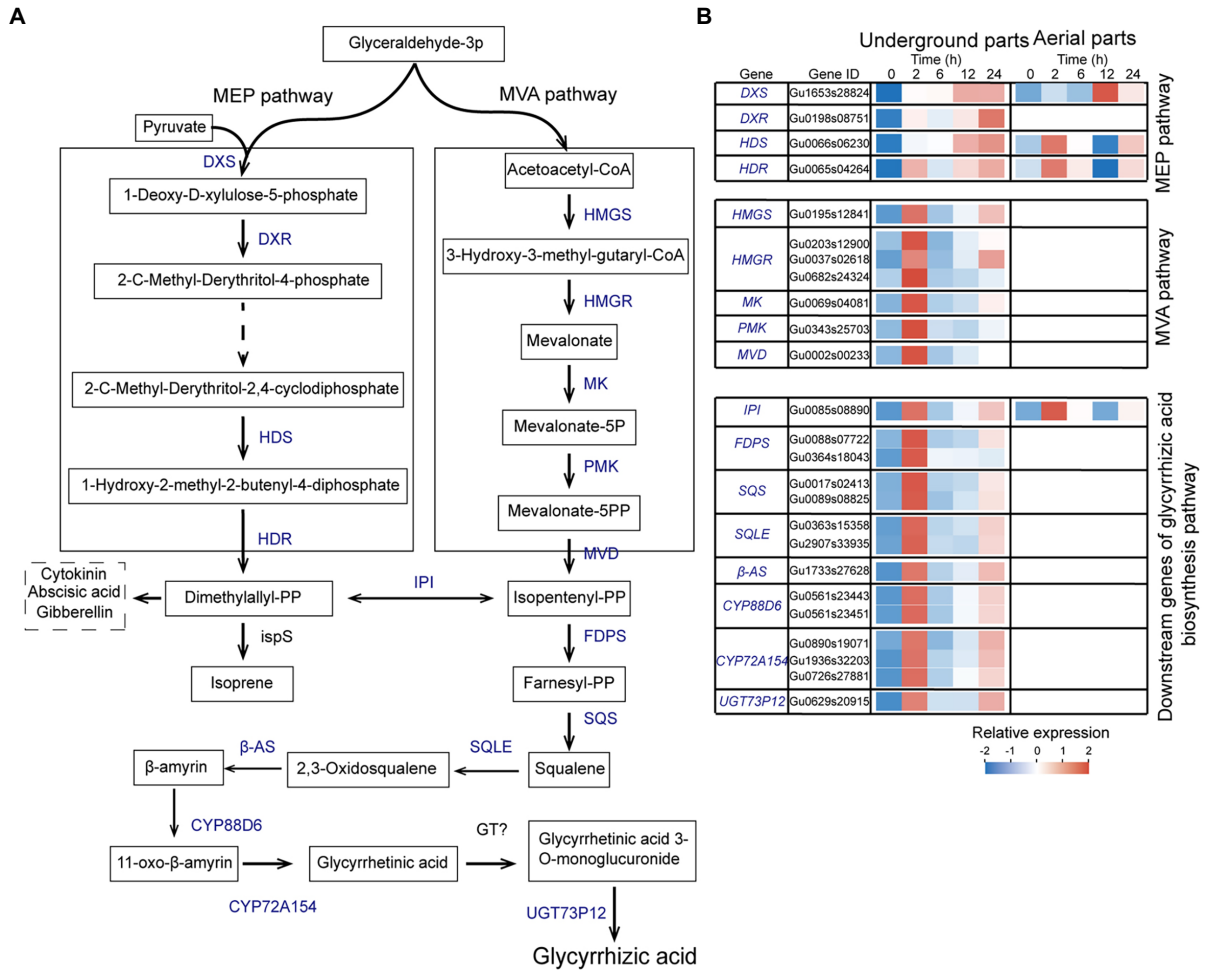
Expression analysis of up-regulated differentially expressed genes (UDEGs) involved in glycyrrhizic acid biosynthesis in *Glycyrrhiza uralensis* under drought stress. **(A)** Identified UDEGs locating in MEP and MVA pathways for glycyrrhizic acid biosynthesis Blue words denote the UDEGs involved in flavonoids biosynthesis of *G. uralensis* under PEG6000-induced drought stress. **(B)** Expression analysis of UDEGs involved in glycyrrhizic acid biosynthesis in *G. uralensis* under drought stress. Different colored blocks showed different expression levels with red and blue to represent high and low levels, respectively. DXS, 1-Deoxy-D-xylulose-5-phosphate synthase; DXR, 1-Deoxy-D-xylulose 5-phosphate reductoisomerase; HDS, 4-Hydroxy-3-methylbut-2-en-1-yl diphosphate synthase; HDR, 4-Hydroxy-3-methylbut-2-enyl diphosphate reductase; IPI, Isopentenyl-diphosphate delta-isomerase; HMGS, Hydroxymethylglutaryl-CoA synthase; HMGR, 3-Hydroxy-3-methylglutaryl-coenzyme A reductase; MK, Mevalonate kinase; PMK, Phosphomevalonate kinase; MVD, Diphosphomevalonate decarboxylase; FDPS, Farnesyl-diphosphate synthase; SQS, Squalene synthase; SQLE, Squalene epoxidase; β-AS, Beta-amyrin synthase; CYP88D6, Beta-amyrin 11-oxidase; CYP72A154, 11-Oxo-beta-amyrin 30-oxidase; UGT73P12, Soyasapogenol B glucuronide galactosyltransferase; ispS, Terpene synthase; GT, Glycosyltransferase.

Glycyrrhizic acid is one of the most important pharmaceutical active ingredients in *G. uralensis*, and its content is an important quality index of licorice. Based on the KEGG enrichment analysis, a total of 25 UDEGs were annotated as encoding enzymes regulating glycyrrhizic acid biosynthesis, with major distribution in underground parts (Figure 4). Mevalonate is the precursor of glycyrrhizic acid biosynthesis by MVA pathway (Li et al., 2020a,b). A total of 21 UDEGs locating in MVA pathway and downstream pathway were identified, including HMGS, *HMGR*, *MK*, *PMK*, *MVD*, *FDPS*, *SQS, SQLE*, *β-AS*, *CYP88D6*, *CYP72A154s*, and *UGT73p12s* that showed similar expression pattern with significant prompt accumulation in 2-h treatment group (Figure 4). In which, *HMGR* (Gu0037s02618), *SQS* (Gu017s00241), and β-AS (Gu1733s27628) displayed 7.5-, 30-, and 70-fold increase expressions in 2-h treatment group compared with that in 0-h control group, respectively (Figure 4; Supplementary Table S8). Expressions of most of these significant enriched UDEGs in aerial parts were low or undetectable (Figure 4; Supplementary Table S9). Meanwhile, as the considered pathway for glycyrrhizic acid synthesis (Hemmerlin et al., 2003; Bartram et al., 2006), MEP pathway and corresponding UDEGs (*DXS*, *DXR*, *HDS*, and *HDR*) were also discovered, with significant induced expression in 12-h and 24-h treatment groups (Figure 4). These results suggest that MVA pathway and corresponding encoding enzymes might perform key function for glycyrrhizic acid synthesis in underground parts of *G. uralensis*.

### Analysis of Endogenous Phytohormone Level and Synthesis and Signal Transduction of JA and ABA Pathway Under Drought Stress

**Figure 5:**
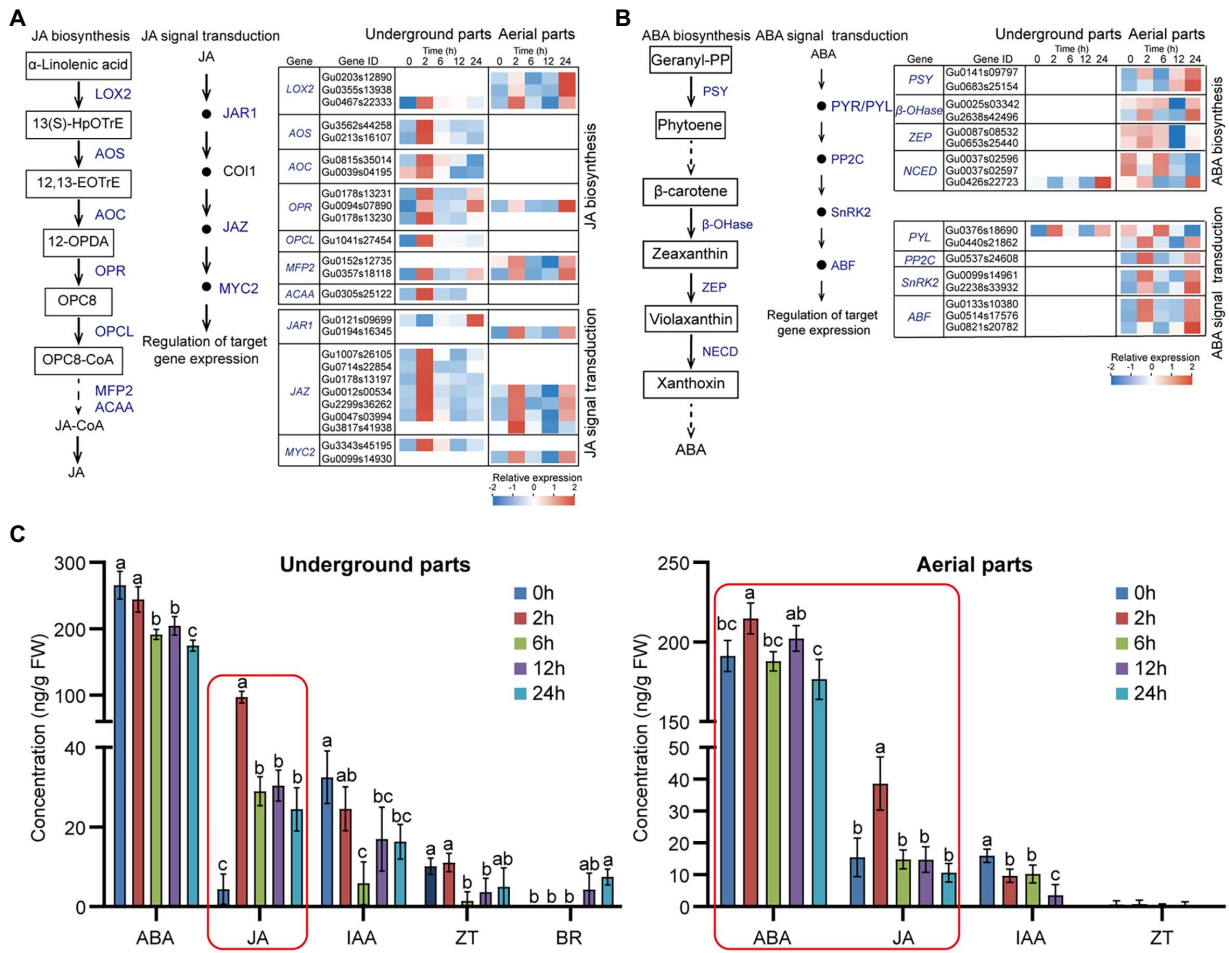
Effects of drought stress on JA and ABA biosynthesis and signal transduction in *Glycyrrhiza uralensis* **(A)** Expression analysis of identified up-regulated differentially expressed genes (UDEGs) locating in JA biosynthesis and signal transduction under drought stress. **(B)** Expression analysis of identified UDEGs involved in ABA biosynthesis and signal transduction under drought stress. **(C)** Detection of endogenous phytohormone levels in aerial and underground parts of *G. uralensis* under drought stress. The main plant hormones including JA, ABA, IAA, ZT, BR, and GA were measured. GA and BR could not be detected in aerial parts of *G. uralensis*, and GA could not be detected in underground parts of *G. uralensis*. Different colored blocks (in **A** and **B**) indicate different expression abundance with red and blue to denote high and low levels, respectively. Blue words represent the UDEGs involved in JA and ABA biosynthesis and signal transduction of *G. uralensis* under drought stress. Red boxes denote the most significantly increased phytohormones in *G. uralensis* under drought stress. Different letters followed by mean ± standard error indicate significant differences at *p* < 0.05 level. 13(S)-HpOTrE, 13(S)-hydroperoxyoctadecatrienoic acid; 12,13-EOTrE, 12,13-Octadecatrienoic acid; 12-OPDA, 12-Oxophytodienoic acid; OPC8, 8-(3-Oxo-2-(pent-2-enyl)cyclopentyl) octanoic acid; LOX2, Lipoxygenase 2; AOS, Allene oxide synthase; AOC, Allene oxide cyclase; OPR, 12-Oxophytodienoate reductase; OPCL, 4-Coumarate-CoA ligase; MEP2, Peroxisomal fatty acid beta-oxidation 2; ACAA, 3-Ketoacyl-CoA thiolase; JAR1, Jasmonic acid-amido synthetase 1; COI1, Coronatine insensitive-1; JAZ, Jasmonate ZIM-domain; MYC2, Transcription factor MYC2; PSY, Phytoene synthase; β-OHase, Beta-carotene hydroxylase; ZEP, Zeaxanthin epoxidase; NCED, 9-Cis-epoxycarotenoid dioxygenase; PYR/PYL, Abscisic acid receptor PYL; PP2C, Phosphatase 2C; SnRK2, Serine/threonine-protein kinase 2; ABF, ABA-responsive element binding factor.

Considering the significant enriched pathways of JA and ABA biosynthesis and signal transduction (Figure 2), to further investigate the effect of drought stress on the expression of genes locating in these pathways, we analyzed the expressions of the UDEGs involved in JA and ABA biosynthesis and signal transduction (Figures 5A,B; Supplementary Table S10). In underground parts, the JA biosynthesis UDEGs including LOX2, allene oxide synthase (AOS), allene oxide cyclase (AOC), 12-oxo-phytodienoic acid reductase 3 (OPR3), OPCL, MFP2, and ACAA indicated consistent expression pattern with significant prompt accumulation in 2-h treatment group (Figure 5A). In which, AOS (Gu3562s44258), AOC (Gu0815s35014), and *OPR3* (Gu0178s13230) showed 15-, 3.2-, and 3.6-fold accumulated expressions in 2-h treatment group compared with that in 0-h control group, respectively (Figure 5A). For the JA signal transduction UDEGs, *JAR1*, *COI1*, *JAZ*, and *MYC2* were discovered, and indicated similar expression feature with the JA biosynthesis UDEGs. In which, six JAZ genes were identified and one (Gu0047s03994) showed significant increase expression with 47.5-fold enrichment in 2-h treatment group than in 0-h control group. In aerial parts, UDEGs of JA biosynthesis and signal transduction showed fewer number and different expression pattern with significant induced accumulation in 2-h and 24-h treatment groups. For the genes involved in ABA biosynthesis and signal transduction, only two UDEGs were observed in underground parts, most of them were discovered in aerial parts including *PSY*, *β-OHase*, *ZEP*, *NCED*, *PYR/PYL*, *PP2C*, *SnRK2*, and *ABF*, and showed significant higher expressions in 2-h and 24-h treatment groups than in 0-h control group (Figure 5B). Two SnRK2 genes (Gu0009s14961 and Gu2238s33932) displayed 15- and 5-fold increase expressions in 2-h treatment group than in 0 treatment group, respectively (Figure 5B; Supplementary Table S6).

Since the UDEGs involved in plant hormone signal transduction pathway were observed, including JA, ABA, IAA, ZT, BR, and GA (Figure 2, Supplementary Table S6), to study the effect of these UDEGs on endogenous phytohormone level, we determined the content of JA, ABA, IAA, ZT, and BR in *G. uralensis* under PEG6000-induced drought stress (Figure 5C). In underground parts, JA content was significantly promoted after 2 h treatment of drought stress and thereafter maintained a high level with over 22-fold increase in 2-h treatment group compared with that in 0-h control group. While, ABA level showed a gradual decrease tendency along with drought treatment. In aerial parts, JA also indicated over 2.5-fold accumulation after 2 h treatment and then decreased as normal level with control. ABA content was slightly increased in 2-h treatment group but not in other groups. The content of other hormones including IAA, BR, and ZT showed relative low levels or could not be detected. These results demonstrated that JA was the most significantly increased phytohormone in both underground and aerial parts of *G. uralensis*, suggesting the possible important role of JA and corresponding genes for *G. uralensis* plants especially the underground parts in response to drought stress.

### Analysis of Levels of Pharmaceutical Active Components in *Glycyrrhiza uralensis* Under Treatment of PEG6000 and JA

**Figure 6:**
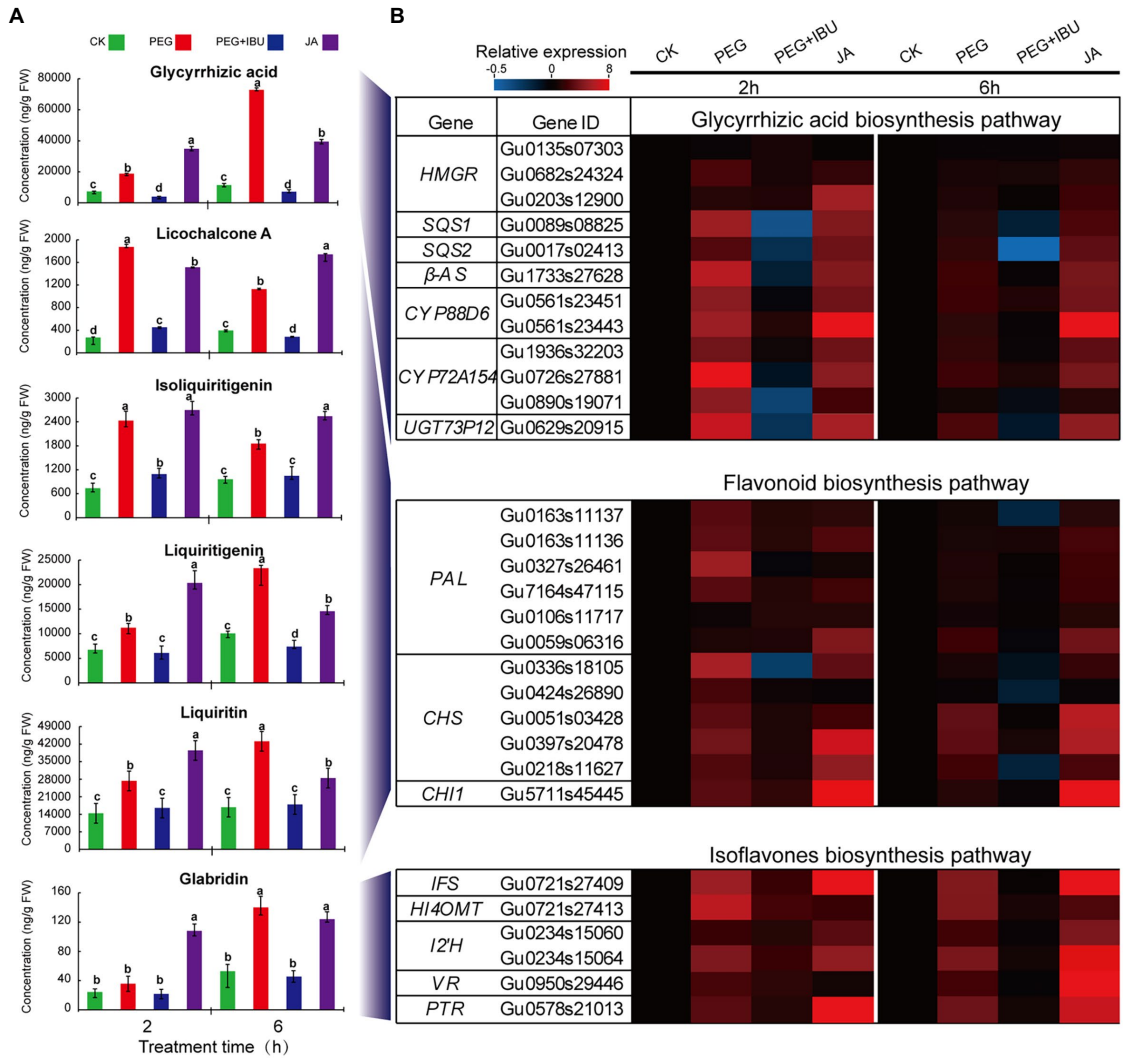
Effects of PEG, JA, and JA synthesis inhibitor IBU on the production of pharmaceutical active components in underground parts of *Glycyrrhiza uralensis*. **(A)** Determination of JA and pharmaceutical active ingredients under treatment of PEG6000 and IBU. Different letters followed by mean ± standard error indicate significant differences at *p* < 0.05 level. **(B)** Expression heatmaps of genes involved in pharmaceutical active ingredient biosynthesis. Water supplement was used as control (CK). Different colored blocks denote different expression levels with red and blue to represent high and low levels, respectively.

Reports indicated that pretreatment of ibuprofen (IBU) to plants inhibited LOX activity, and thus decreased JA content and JA-dependent downstream products (Staswick et al., 1991; Zhang et al., 2009; Mandal et al., 2015). Considering the results of the most significant increase of JA content in underground parts under PEG6000 treatment, thus, to investigate the function of JA in regulating pharmaceutical active components, JA, PEG6000, and mix of PEG6000 and IBU were used to treat *G. uralensis* underground parts to determine these ingredients. The results showed that, liquiritin, liquiritigenin, isoliquiritigenin, glycyrrhizic acid, licochalcone A, and glabridin displayed prompt accumulation after 2 and 6 h treatment of PEG6000 and JA. The addition of mix of PEG6000 and IBU significantly decreased the content of these pharmaceutical active components (Figure 6A). The expressions of the identified UDEGs encoding the enzymes involved in the synthesis of pharmaceutical active components indicated similar features that were induced under treatment of PEG6000 and JA and were inhibited under treatment of IBU through qRT-PCR assays (Figure 6B). In the treated *G. uralensis* aerial parts, similar results were also observed with a significant low content of these pharmaceutical active components than in underground parts (Supplementary Figure S4). These results indicated that both PEG6000-induced drought stress and JA are the key factors to stimulate the production of pharmaceutical active components, especially in the underground parts of *G. uralensis*.

### Effect of PEG6000-Induced Drought Stress on JA Content in *Glycyrrhiza uralensis*

**Figure 7:**
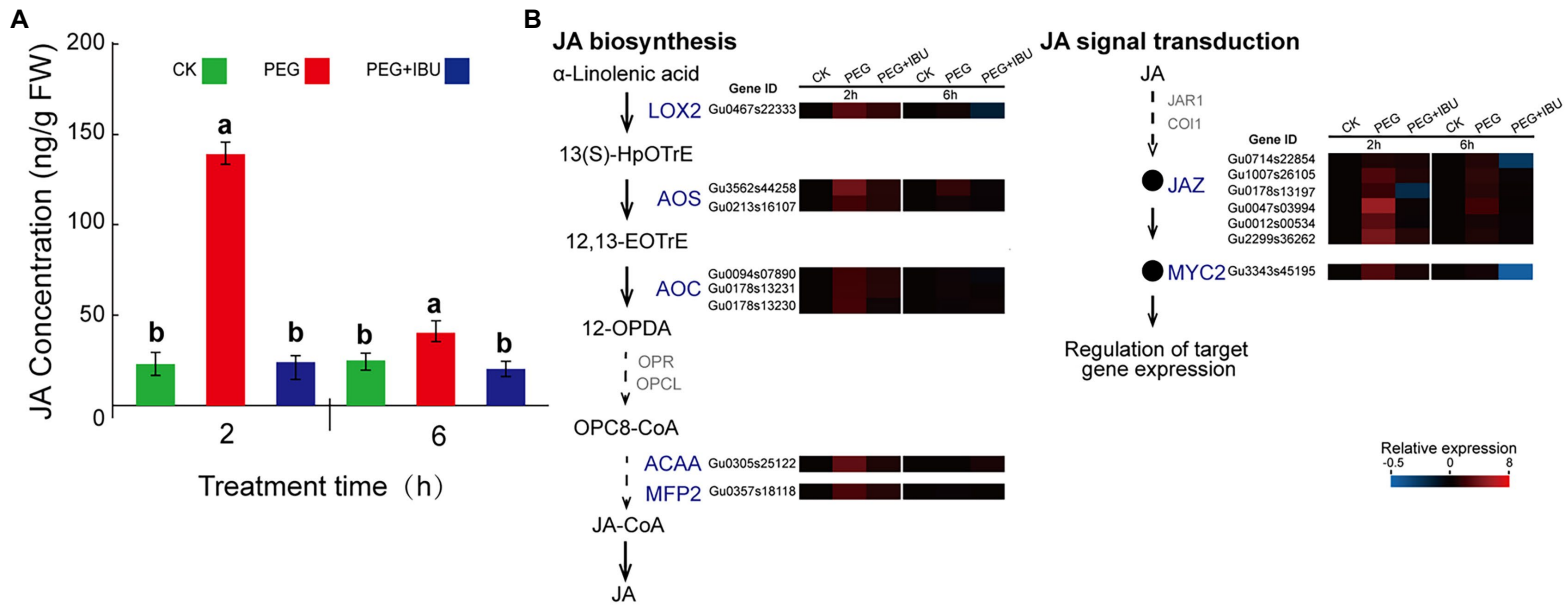
Effect of PEG6000 on JA synthesis and signal transduction in underground parts of *Glycyrrhiza uralensis*. **(A)** Determination of JA content under treatments of PEG6000 and mix of PEG6000 and IBU. Different letters followed by mean ± standard error indicate significant differences at *p* < 0.05 level. **(B)** Expression heatmaps of genes involved in JA biosynthesis and signal transduction under treatments of PEG6000 and mix of PEG6000 and IBU. Water addition was used as control (CK). Different colored blocks denote different expression levels with red and blue to represent high and low levels, respectively.

To study the connection between drought stress and JA synthesis and signal transduction, PEG6000, and mix of PEG6000 and IBU were used to treat *G. uralensis*, to determine the levels of JA content and expressions of its biosynthesis and signal transduction genes. The results showed that JA content in underground parts was significantly increased under 2 h treatment of PEG6000, and decreased as normal level with non-treated control (Figure 7A). At the same time, the genes encoding JA biosynthesis of *LOX2*, *AOS*, *AOC*, *ACAA*, and *MFP2* and signal transduction of *JAZ* and *MYC2*, indicated same expression features of increased expression by PEG6000 and decreased expression by IBU (Figure 7B). In the PEG6000-treated *G. uralensis* aerial parts, JA content and gene expression levels also indicated similar results but with a relative low levels than in underground parts (Supplementary Figure S4). These results demonstrate that PEG6000 has a direct positive effect to stimulate JA production and signal transduction.

## Discussion

Secondary metabolites such as glycyrrhizic acid and flavonoids are considered to play important function in response to abiotic stress (Afreen et al., 2005; Hayashi and Sudo, 2009). The yield of valuable compounds can be improved by drought-mediated induction of aromatic products, such as isoprenoids, phenols, and alkaloids in medicinal and spice plants (Selmar and Kleinwachter, 2013; Kleinwachter and Selmar, 2015). Moderate drought stress has positive role to stimulate the production of pharmaceutical active ingredients in *G. uralensis* (Li et al., 2011), and our previous studies indicated that salt stress promoted the accumulation of glycyrrhizic acid and flavonoids (Tong et al., 2019). In this study, we found that PEG6000-induced drought stress significantly promotes the accumulation of pharmaceutical active ingredients, including glycyrrhizic acid, liquiritin, isoliquiritigenin, liquiritigenin, licochalcone-A, and glabridin, in *G. uralensis* with a major effect in the underground parts (Figure 1). The results also indicated that glycyrrhizic acid content displayed a continuous increase after PEG treatment, whereas the contents of the other substances of liquiritin, isoliquiritigenin, liquiritigenin, licochalcone-A, and glabridin indicated significant accumulation after 2 h treatment of PEG, with the overall tendency of raise first and then decrease (Figure 1). Secondary metabolites such as flavonoids compounds and terpenoids indicated a rapid induction and then reduction in plants response to abiotic stress acting as antioxidant activity (Cao et al., 1997; Rezaie et al., 2020; Wang et al., 2020; Zhu et al., 2020a,b), providing the important role in eliminating ROS to maintain the cellular oxidoreduction homeostasis (Wang et al., 2020).

Glycyrrhizic acid is one of the most abundant pharmaceutical active ingredients in the underground parts of *G. uralensis*, and was synthesized from mevalonate, catalyzed by a series of enzymes encoded by corresponding synthesis genes and downstream genes (Haralampidis et al., 2002; Seki et al., 2008, 2011; Li et al., 2017; Mochida et al., 2017; Nomura et al., 2019). In this work, through RNA-seq analysis, genes of *β-AS, CYP88D6*, *CYP72A154*, and *UGT73P12* were significantly up-regulated in *G. uralensis* induced after PEG6000 treatment (Figures 2, 4). β-AS is a key enzyme in the glycyrrhizic acid biosynthesis by catalyzing the conversion of 2,3-oxidosqualene into β-amyrin and is responsible for the triterpene skeleton formation (Hayashi et al., 2001; Wang et al., 2011). Glycosyltransferase UGT73P12 transfers the glucuronic part of UDP-glucuronic acid to glycyrrhetinic acid 3-O-monoglucuronide to produce glycyrrhizic acid (Nomura et al., 2019).

Flavonoids in licorice are the main physiological pharmaceutical active ingredients that play important role in resisting abiotic stress and promoting plant growth (Yang et al., 2007; Li et al., 2009). In the pathway governing flavonoids biosynthesis, many genes have been investigated to show significant up-regulation, including *PAL*, *ACC*, *C4L*, *4CL*, *CHS*, *II-CHI*, *IFS*, *HI4OMT*, *HIDH*, *I2′H*, *VR,* and *PTR*. High expression of *4CL*, *ACC*, *CHS*, *II-CHI*, and *IFS* may contribute to the accumulation of flavonoids and isoflavones (Shimada et al., 2003; Fatland et al., 2005; Tan et al., 2012; Yin et al., 2019). In the present work, a total of 21 UDEGs locating in flavonoids synthesis pathway in *G. uralensis* were identified under drought stress (Figure 3; Supplementary Table S7). In which, seven GuCHS indicated significant increased expressions and tissue-specific feature with five in underground parts and two in aerials parts, respectively (Figures 2, 3). CHS and II-CHI were considered to be the key rate-limiting enzymes in the liquiritin synthesis pathway in *G. uralensis* (Shimada et al., 2003; Yin et al., 2019). Studies also showed that different *CHS* genes usually have different expression patterns (Harker et al., 1990; Han et al., 2006; Qiao et al., 2015), suggesting the potential diverse functions of the CHSs. Glabridin is an unique active ingredient in licorice, and is considered to be synthesized in the isoflavone synthesis pathway using IFS as key enzyme (Jung et al., 2000; Simmler et al., 2013). We found that glabridin content and *IFS* expression level were significantly promoted in both aerial and underground parts of *G. uralensis* under drought stress (Figures 1–3), implying the possible role of *IFS* in glabridin biosynthesis.

Phytohormones are key regulators to play important role for plants in response to abiotic stress by generating many secondary metabolites (Bari and Jones, 2009; Nakashima and Yamaguchi-Shinozaki, 2013). JA and ABA regulated the synthesis of flavonoids and triterpenoids by activating MYC2 transcription factors during drought and salt stress (Sreenivasulu et al., 2012). Our study showed the consistent result that the content of JA in aerial and underground parts and ABA in aerial parts were significantly increased after PEG6000-induced drought stress (Figure 5C). It is established that ABA biosynthesis could occur *via* carotenoid biosynthesis pathway (Iuchi et al., 2001; Seo and Koshiba, 2002). We found that the UDEGs of ABA biosynthesis and signal transduction were significantly identified in aerial parts (Figures 2, 5). Some reports support that SnRK2 in the ABA signaling pathway plays an important role in regulating flavonoid synthesis in plants under abiotic stress (Boudsocq et al., 2004; Kobayashi et al., 2004; Jiao and Gu, 2019). There is also solid evidence that SnRK2 and ABF performed important function in controlling the downstream gene expressions under abiotic stress (Fujii et al., 2011; Kulik et al., 2011; Fujita et al., 2013; Yoshida et al., 2015; Fatima et al., 2020). SnRK2 and ABF were identified as the most significant enriched UDEGs and showed high expression levels under drought stress (Figures 2, 5), suggesting their potential important function in ABA signal transduction and regulation of secondary compound generation.

JA biosynthesis could produce through α-linolenic acid metabolic pathway (Wasternack and Hause, 2013). α-linolenic acid metabolism pathway was significantly enriched in both aerial and underground parts in *G. uralensis* (Figure 2). And many genes locating in this pathway were identified as UDEGs regulating JA biosynthesis and signal transduction (Figure 5), including LOX2, AOS, AOC, OPR, JAZ, and MYC2, etc., providing a close direct link between drought stress and JA signaling. AOS, AOC, and OPR3 have been reported to be the key enzymes involved in JA biosynthesis in plants (Wasternack and Hause, 2013). JAZ and MYC2 are important regulators to modulate JA signal transduction (Chini et al., 2007; Liu et al., 2019). Under abiotic stress, MYC2, acting as a master regulator of diverse aspects of JA responses (Boter et al., 2004; Lorenzo et al., 2004; Dombrecht et al., 2007; Du et al., 2017), regulated the specific production of defense-related metabolites, including terpenoids and flavonoids, to protect plants against stress (Tropf et al., 1995; Jung et al., 2000; Ogawa et al., 2017; Yang et al., 2017; Premathilake et al., 2020). In the present study, genes involved in glycyrrhizic acid and flavonoids synthesis were inhibited by JA biosynthesis inhibitor IBU (Figure 6), and addition of both PEG6000 and JA significantly promoted the accumulation of glycyrrhizic acid and flavonoids content (Figure 6A), as well as the increase of expression levels of corresponding genes (Figure 6B). PEG6000-induced drought stress also generated a positive role for JA production and corresponding gene expressions (Figure 7), suggesting a direct close connection between drought stress and JA synthesis and signaling and thus to promote the accumulation of pharmaceutical active ingredients. As far as the difference of JA and ABA being concerned, the results showed that, contents of JA in aerial and underground parts, and the expressions of key genes involved in JA synthesis and signaling were significantly increased by PEG6000-induced drought stress (Figures 5, 6), with the JA content being over 22-fold increase in underground parts and over 2.5-fold accumulation in aerial parts in 2-h treatment group compared with that in 0-h control group (Figure 5). Meantime, both ABA content and UDEGs of ABA biosynthesis and signal transduction were significantly raised in aerial parts (Figures 2, 5), but not in underground parts, with ABA content being a slight increase of approximate 1.12 fold in aerial parts (Figure 5). Therefore, JA and its mediated signaling could be selected as important target candidates for further utilization.

In conclusion, through a combined comprehensive analysis of pharmaceutical active ingredients and comparative transcriptomes, our results indicated that PEG6000-induced drought stress could significantly promote the accumulation of pharmacological active ingredients in both aerial and underground parts of *G. uralensis*, *via* the JA-mediated signaling pathway. Our study also offers the candidate key genes and metabolic pathways of glycyrrhizic acid and flavonoids in *G. uralensis* under drought stress, and provides effective references for genetic improvement and comprehensive utilization of *G. uralensis*, with regulation mechanism elucidation and genetic function analysis of these key genes to be warranted.

## Data Availability Statement

The datasets presented in this study can be found in online repositories. The names of the repository/repositories and accession number(s) can be found at: https://www.ncbi.nlm.nih.gov/, PRJNA810509.

## Author Contributions

HY: software, investigation, and data curation. FW: investigation, validation, and formal analysis. QB: methodology, software, and investigation. HL: visualization. LL: collection and identification of *G. uralensis* resources. GX: conceptualization. JZ: conceptualization and supervision. HS: conceptualization, resources, writing—original draft preparation, supervision, project administration, and funding acquisition. HL: conceptualization, writing—review and editing, supervision, project administration, and funding acquisition. All authors contributed to the article and approved the submitted version.

## Funding

This work was supported by Science and technology project of Bingtuan (grant numbers 2018AB012 and 2020AA005), Scientific research project of Shihezi University (grant numbers ZZZC201931B, RCZK2021B31, and YZZX202102).

## Conflict of Interest

The authors declare that the research was conducted in the absence of any commercial or financial relationships that could be construed as a potential conflict of interest.

## Publisher’s Note

All claims expressed in this article are solely those of the authors and do not necessarily represent those of their affiliated organizations, or those of the publisher, the editors and the reviewers. Any product that may be evaluated in this article, or claim that may be made by its manufacturer, is not guaranteed or endorsed by the publisher.

## Footnotes

^1^ https://github.com/jstjohn/SeqPrep

^2^ https://github.com/najoshi/sickle

^3^ http://trinityrnaseq.sourceforge.net/

^4^ http://ccb.jhu.edu/software/tophat/index.shtml

^5^ http://ccb.jhu.edu/software/hisat2/index.shtml

^7^ http://deweylab.biostat.wisc.edu/rsem/

^8^ http://www.bioconductor.org/packages/2.12/bioc/html/edgeR.html

^9^ http://kobas.cbi.pku.edu.cn/home.do
