# Supplementary material for: Combined Analysis of Pharmaceutical Active Ingredients and Transcriptomes of Glycyrrhiza uralensis Under PEG6000-Induced Drought Stress Revealed Glycyrrhizic Acid and Flavonoids Accumulation via JA-Mediated Signaling
Source: Front Plant Sci. 2022 Jun 13;13:920172. doi: 10.3389/fpls.2022.920172 (PMC9234494; doi:10.3389/fpls.2022.920172)
Supplement: Supplementary file 1 [file Data_Sheet_1.ZIP › Supp Figures and Tables/Supplementary Figures S1-S4 submit 20220226.docx]

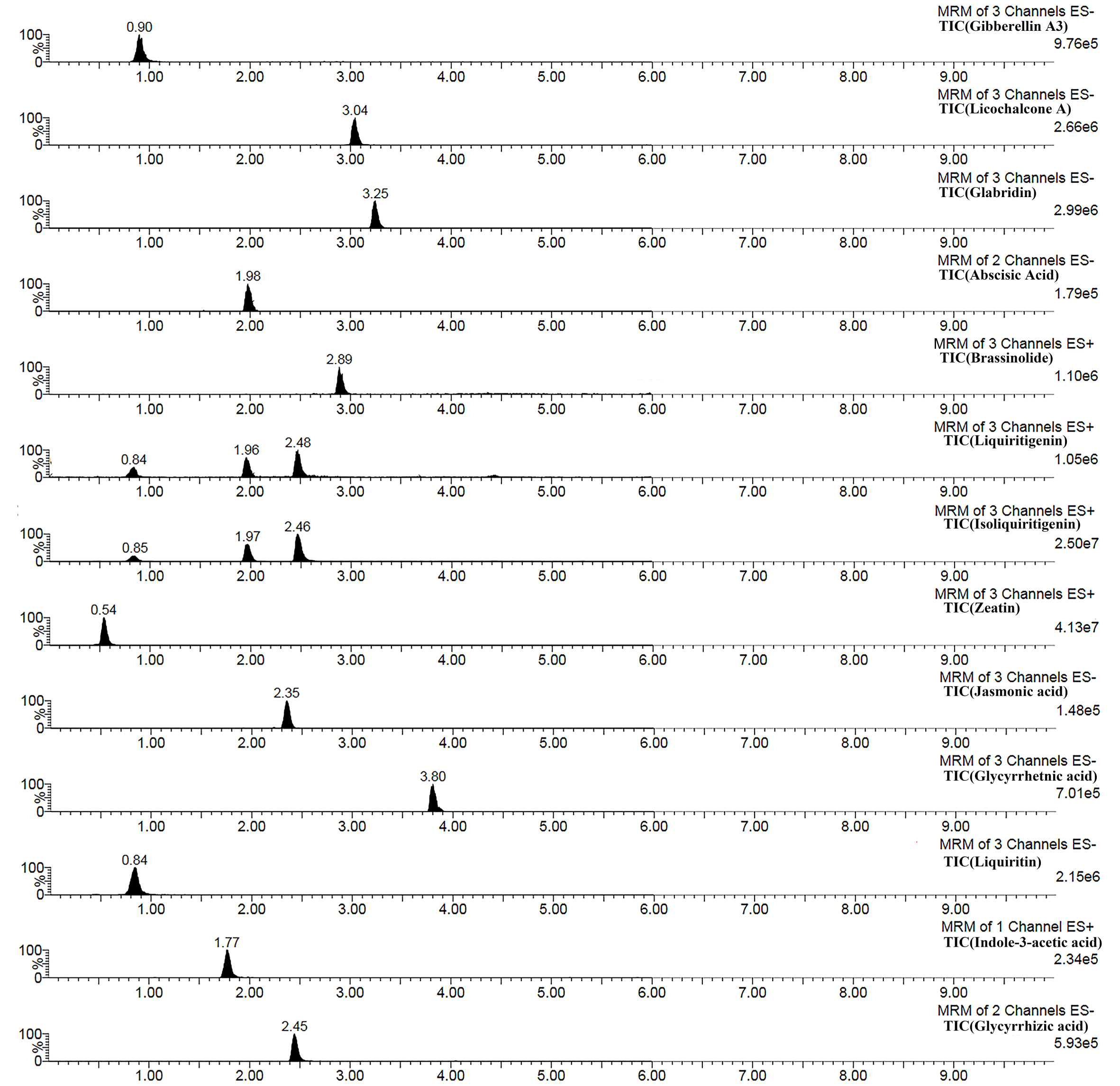


**Supplementary Figure S1.** Total ion flow (TIC) chromatogram of the standards of the 13 detected substances.


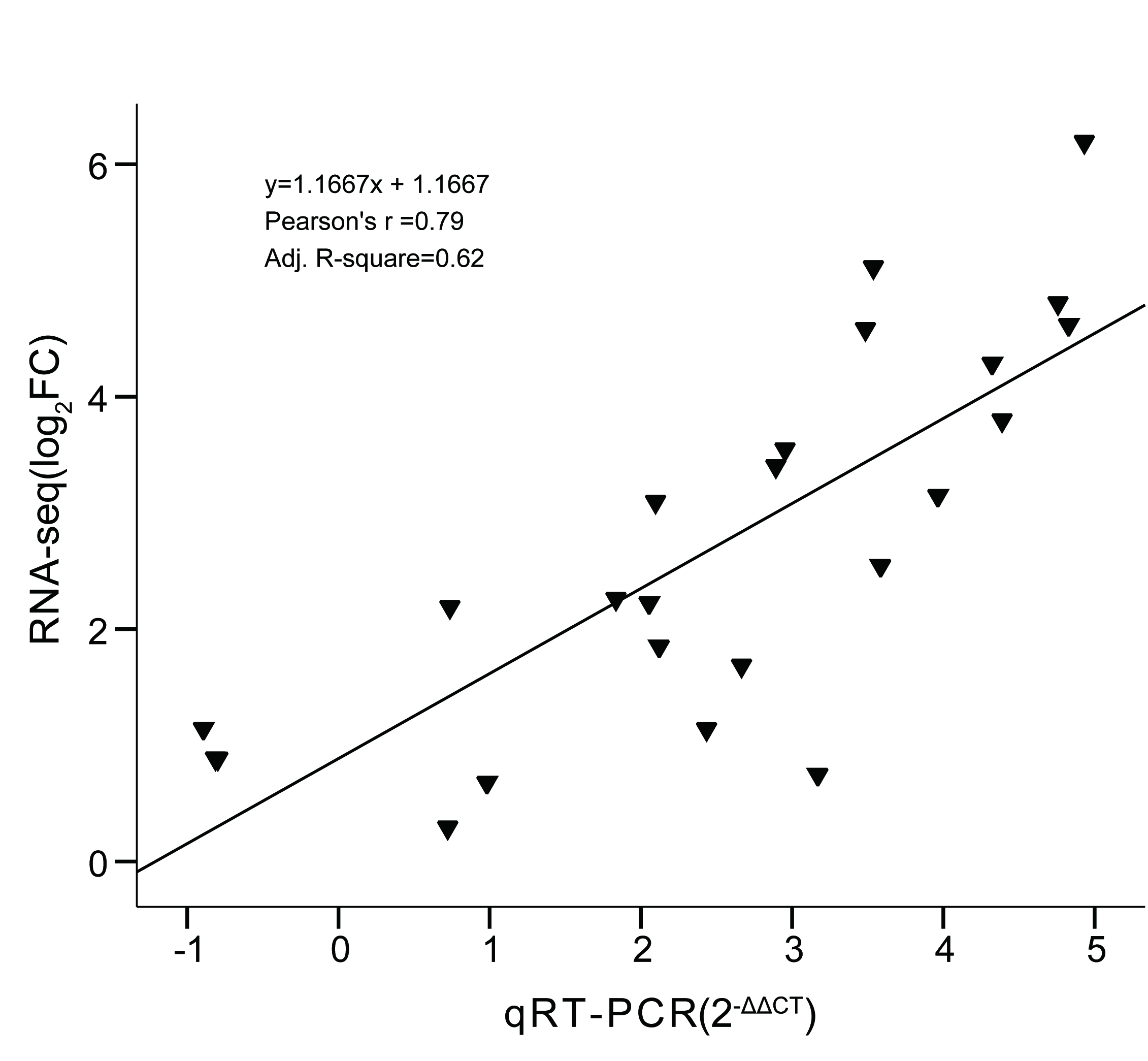


**Supplementary Figure S2.** Validation of RNA-Seq ata by qRT-PCR.


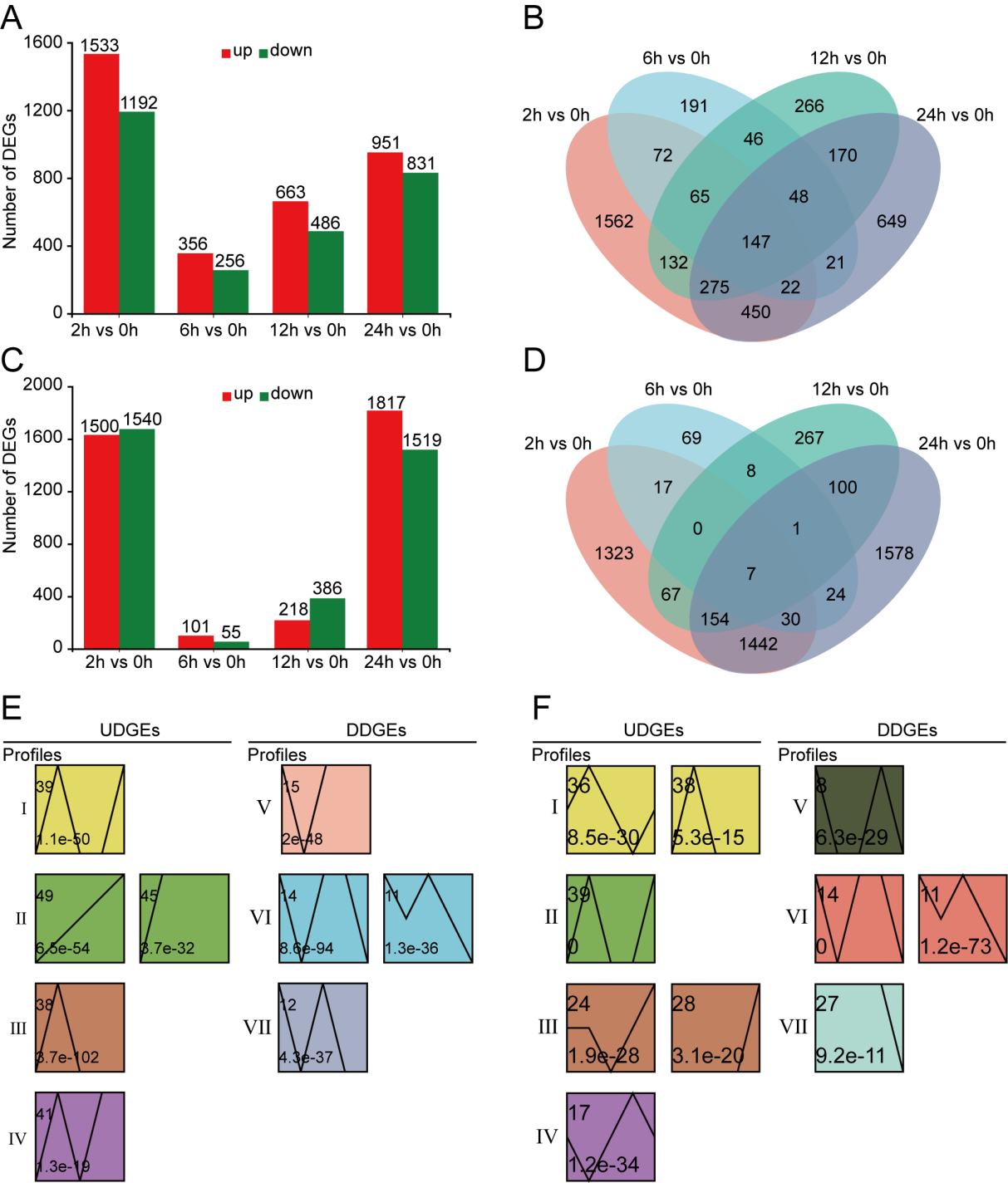


**Supplementary Figure S3.**  Transcriptome analysis of *G. uralensis* under PEG stress at different ties. Sixty-day-old *G. uralensis* plants treated by 10% PEG6000 for 0h, 2h, 6h, 12h and 24h were collected for generation of RNA-sequencing (RNA-seq) data, and the identified DEGs were used for further analysis. (A) Statistics analysis of the DEGs identified in different treatment time points under PEG stress in the underground parts. (B) Venn diagram analysis of the DEGs in the three comparison groups at different time of PEG treatment in the underground parts. (C) Statistics analysis of the DEGs identified in different treatment time points under PEG stress in the aerial parts. (D) Venn diagram analysis of the DEGs in the three comparison groups at different time of PEG treatment in the aerial parts. (E) Temporal expression pattern analysis of the up-regulated DEGs(UDGEs) and down-regulated DEGs(DDGEs) in the underground parts. (F) Temporal expression pattern analysis of the up-regulated DEGs (UDGEs) and down-regulated DEGs (DDGEs) in the aerial parts. In the Short Time-Series Expression Miner (STEM), different color clusters represent different profiles.


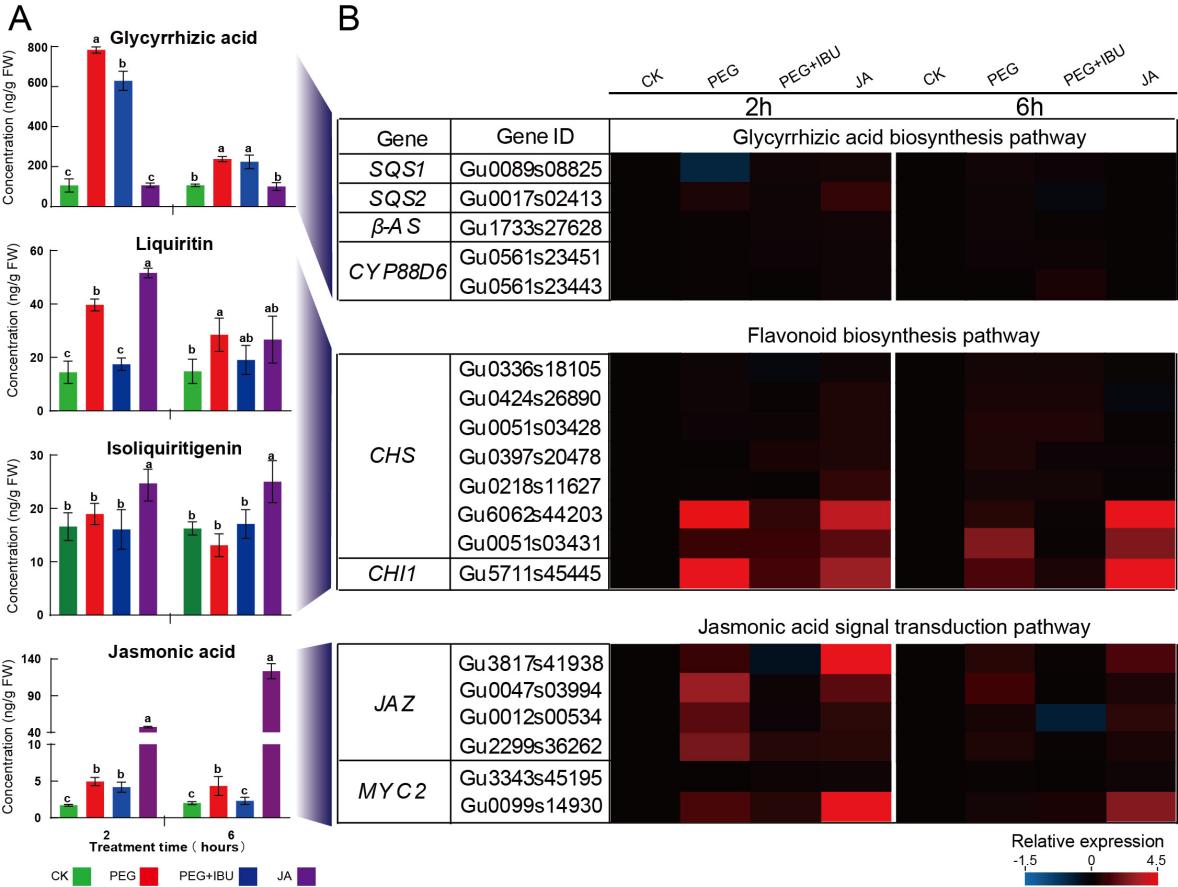


**Supplementary Figure S4.** Effects of PEG and JA synthesis inhibitor IBU on the production of pharmaceutical active components in aerial parts of *G. uralensis*.

(A) Determination of JA and pharmaceutical active ingredients under treatment of PEG and IBU. Different letters followed by mean ± standard error indicate significant differences at p < 0.05 level. (B) Expression heatmaps of genes involved in pharmaceutical active ingredient biosynthesis and JA biosynthesis and signal transduction. Different colored blocks denote different expression levels with red and blue to represent high and low levels respectively.
