# Supplementary figures and images for: Combined Analysis of Pharmaceutical Active Ingredients and Transcriptomes of Glycyrrhiza uralensis Under PEG6000-Induced Drought Stress Revealed Glycyrrhizic Acid and Flavonoids Accumulation via JA-Mediated Signaling
Source: Front Plant Sci. 2022 Jun 13;13:920172. doi: 10.3389/fpls.2022.920172 (PMC9234494; doi:10.3389/fpls.2022.920172)

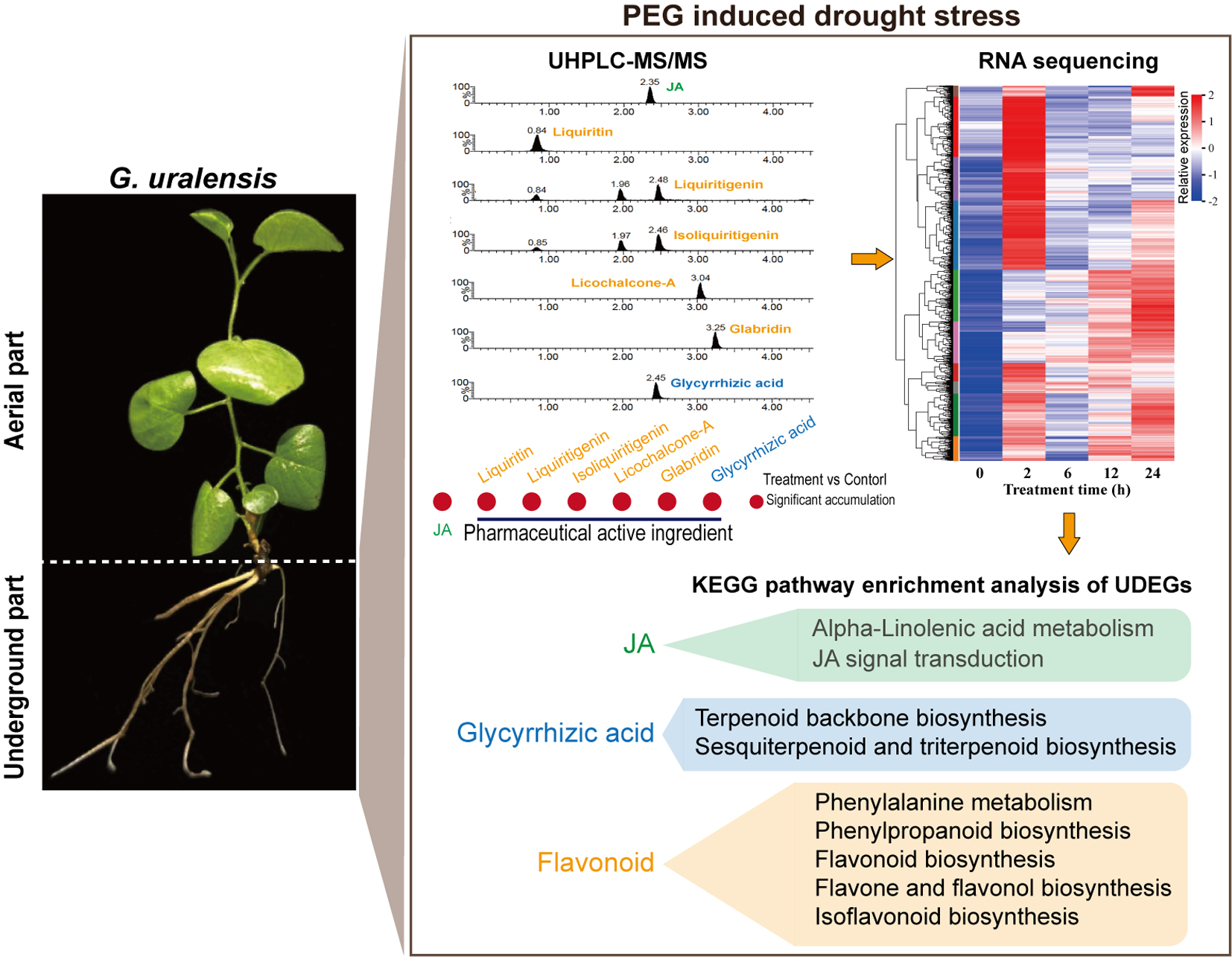

Supplement: Supplementary file 2 [file Image_1.JPEG]
